# Supplementary material for: Melt-Spun Nanocomposite Fibers Reinforced with Aligned Tunicate Nanocrystals
Source: Polymers (Basel). 2019 Nov 20;11(12):1912. doi: 10.3390/polym11121912 (PMC6960881; doi:10.3390/polym11121912)
Supplement: Supplementary file 1 [file polymers-11-01912-s001.pdf]

Melt-Spun Nanocomposite Fibers Reinforced with Aligned Tunicate Nanocrystals

–Supplementary Information –

Alexandre Redondo [1], Sourav Chatterjee [2], Pierre Brodard [3], LaShanda T.J. Korley [2,4], Christoph Weder [1], Ilja Gunkel [1], Ullrich Steiner [1,*]

[1] Adolphe Merkle Institute, University of Fribourg, Chemin des Verdiers 4, 1700 Fribourg, Switzerland
[2] Department of Materials Science and Engineering, University of Delaware, Newark, Delaware 19716, United States
[3] University of Applied Sciences of Western Switzerland, College of Engineering and Architecture of Fribourg, Boulevard de Pérolles 80, CH-1705 Fribourg, Switzerland
[4] Department of Chemical and Biomolecular Engineering, University of Delaware, Newark, Delaware 19716, United States

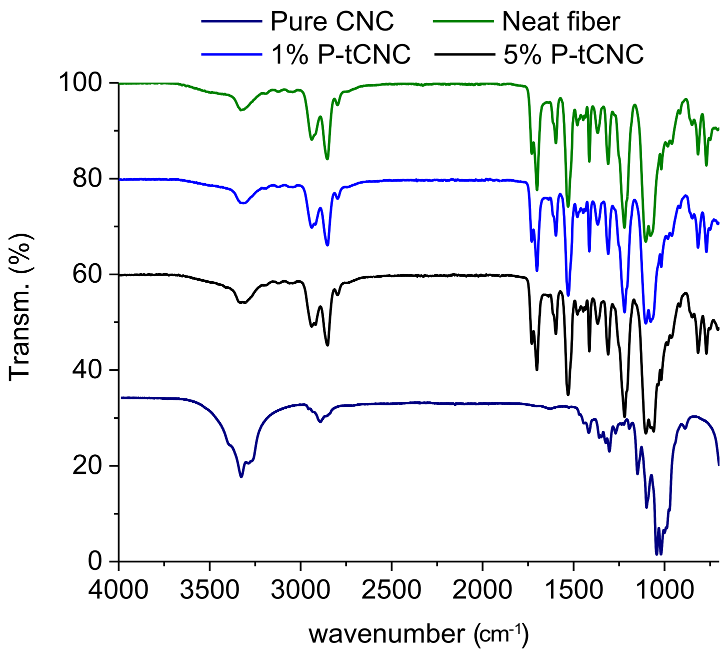

Figure S1: FT-IR spectra of 0, 1 and, 5 wt% P-tCNC melt-spun fibers and neat P-tCNC films. The N-H stretching region at 3000-3500 cm^{-1} can be observed in the spectrum of the neat PU fiber. Upon CNC addition, this peak is seen to change due to the contribution of the CNC OH-groups overlapping with the N-H vibration.

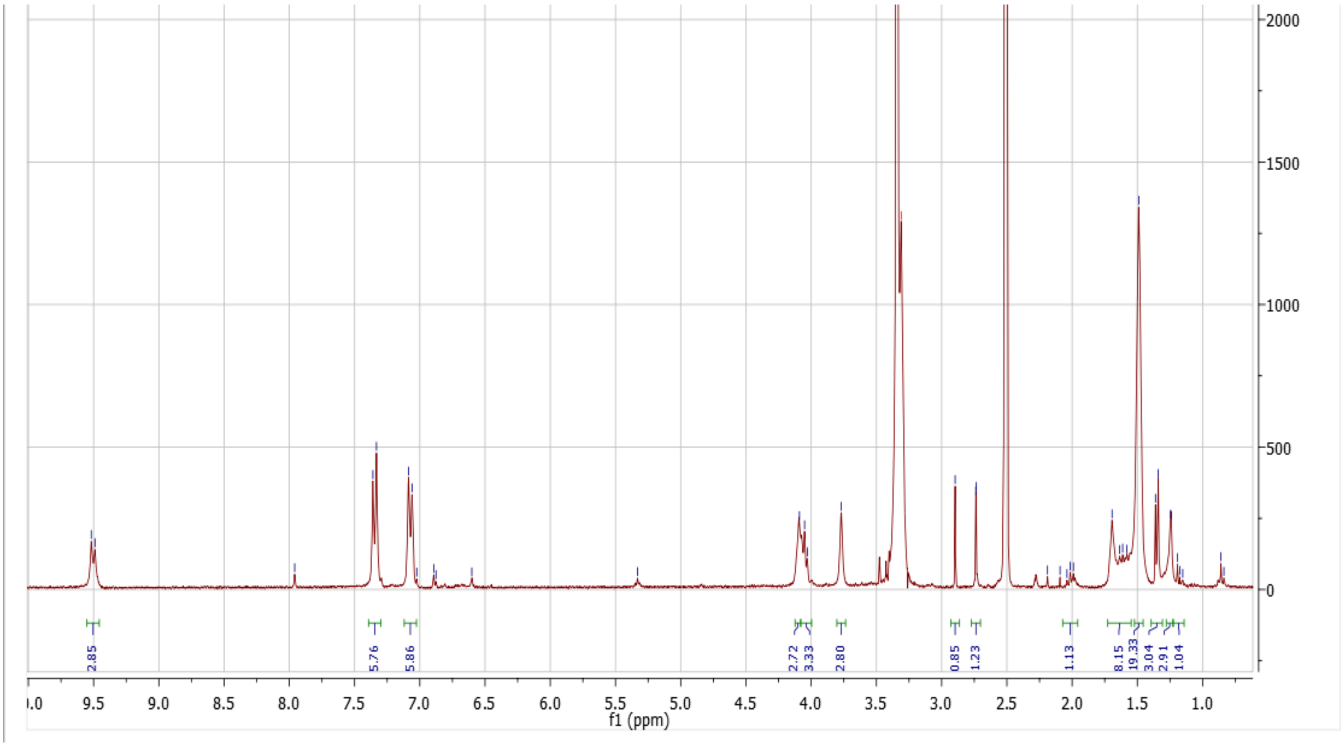

Figure S2: ^1H NMR spectrum of Texin 985 indicating the presence of the urethane N-H at 9.5 ppm, the aromatic protons at 6.5-8 ppm, and the proton near the amide and ether bond at 2.5-4.5 ppm.

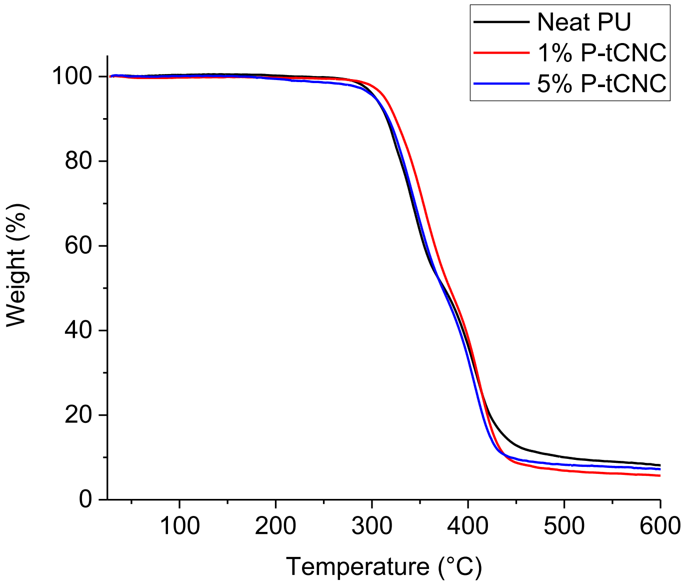

Figure S3: TGA traces of melt-spun fibers containing 0, 1 and 5 wt% P-tCNCs confirming their thermal stability irrespective of the P-tCNC concentration.

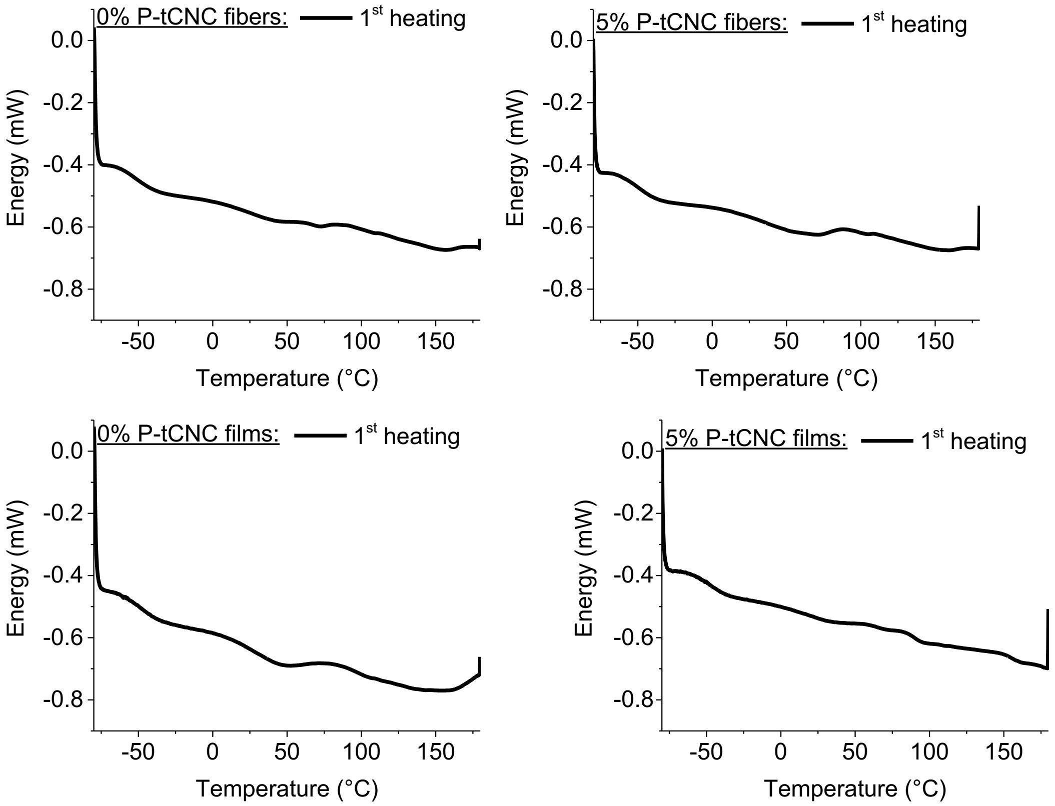

Figure S4: DSC first heating curves of neat PU and 5 wt% P-tCNC nanocomposite fibers and films indicating slight differences in their thermal history due to differences in processing between melt-spun fibers and solvent-cast films.
